# Mortality in women given diethylstilbestrol during pregnancy

**DOI:** 10.1038/sj.bjc.6603221

**Published:** 2006-06-20

**Authors:** L Titus-Ernstoff, R Troisi, E E Hatch, J R Palmer, L A Wise, W Ricker, M Hyer, R Kaufman, K Noller, W Strohsnitter, A L Herbst, P Hartge, R N Hoover

**Affiliations:** 1 Department of Community and Family Medicine, Dartmouth Medical School, and the Norris Cotton Cancer Center, Lebanon, NH 03756, USA; 2 Division of Cancer Epidemiology and Genetics, National Cancer Institute, Bethesda, MD 20892, USA; 3 Department of Epidemiology and Biostatistics, Boston University School of Public Health, Boston, MA 02118, USA; 4 Slone Epidemiology Center, Boston University School of Public Health, Boston, MA 02215, USA; 5 Information Management Services, Rockville, MD 20852, USA; 6 Department of Obstetrics and Gynecology, Methodist Hospital, Houston, TX 77030, USA; 7 Department of Obstetrics and Gynecology, New England Medical Center, Boston, MA 02111, USA; 8 Department of Obstetrics and Gynecology, University of Chicago, Chicago, IL 60637, USA

**Keywords:** DES, oestrogens, breast cancer, mortality

## Abstract

We used Cox regression analyses to assess mortality outcomes in a combined cohort of 7675 women who received diethylstilbestrol (DES) through clinical trial participation or prenatal care. In the combined cohort, the RR for DES in relation to all-cause mortality was 1.06 (95% CI=0.98–1.16), and 1.11 (95% CI=1.02–1.21) after adjusting for covariates and omitting breast cancer deaths. The RR was 1.07 (95% CI=0.94–1.23) for overall cancer mortality, and remained similar after adjusting for covariates and omitting breast cancer deaths. The RR was 1.27 (95% CI=0.96–1.69) for DES and breast cancer, and 1.38 (95% CI=1.03–1.85) after covariate adjustment. The RR was 1.82 in trial participants and 1.12 in the prenatal care cohort, but the DES–cohort interaction was not significant (*P*=0.15). Diethylstilbestrol did not increase mortality from gynaecologic cancers. In summary, diethylstilbestrol was associated with a slight but significant increase in all-cause mortality, but was not significantly associated with overall cancer or gynaecological cancer mortality. The association with breast cancer mortality was more evident in trial participants, who received high DES doses.

Starting in about 1940, diethylstilbestrol (DES), a powerful nonsteroidal oestrogen, was used to prevent pregnancy loss and complications. Although clinical trials conducted in the 1950s showed DES was not effective (Dieckmann *et al*, 1953; Swyer and Law, 1954), use continued for another two decades. Diethylstilbestrol was withdrawn from use during pregnancy in the early 1970s, after it was shown that *in utero* exposure was strongly associated with the risk of vaginal adenocarcinoma (Herbst *et al*, 1971). Until that time, as many as 2 million women in the US (Noller and Fish, 1974), and 4 million women worldwide (Newbold, 1993) were given DES during pregnancy.

Most studies, but not all (Vessey *et al*, 1983), have suggested a positive association between DES taken during pregnancy and breast cancer incidence (Bibbo *et al*, 1978; Clark and Portier, 1979; Beral and Colwell, 1980; Greenberg *et al*, 1984; Hadjimichael *et al*, 1984; Colton *et al*, 1993; Titus-Ernstoff *et al*, 2001), although the latter four studies produced findings compatible with chance. Of the studies assessing DES in relation to breast cancer mortality, one observed a significant association (Calle *et al*, 1996); four produced suggestive findings that were not of statistical significance (Bibbo *et al*, 1978; Clark and Portier, 1979; Hadjimichael *et al*, 1984; Colton *et al*, 1993); and another found no association (Greenberg *et al*, 1984). To date, only one study formally assessed all-cause or disease-specific mortality outcomes, and found little evidence of an association with DES (Greenberg *et al*, 1984). In the present paper, we present findings based on the largest study to date of mortality outcomes in women with documented DES exposure.

## MATERIALS AND METHODS

We assessed exposure to DES during pregnancy in relation to cause-specific and total mortality, with a particular interest in gynaecological cancers, and those known to be influenced by exposure to exogenous oestrogen. The analyses were based on a combined cohort, the design and methods of which have been described previously (Titus-Ernstoff *et al*, 2001).

The combined cohort consists of DES-exposed and unexposed women from two previous follow-up studies, the Women's Health Study (WHS) and the Dieckmann Study, both of which assessed the long-term health consequences of DES exposure during pregnancy. The WHS enrolled DES-exposed and unexposed women who were ascertained through a retrospective review of obstetrics records for the period 1940–1960 at the Mayo Clinic in Rochester, MN; Mary Hitchcock Memorial Hospital in Hanover, NH; a pregnancy clinic at the Boston Lying-In Hospital, in Boston, MA; and a private obstetrics practice in Portland, ME (Greenberg *et al*, 1984; Colton *et al*, 1993; Titus-Ernstoff *et al*, 2001). Diethylstilbestrol-exposed women were those whose records indicated that DES (or, rarely, another nonsteroidal oestrogen) had been prescribed during at least one pregnancy resulting in a live birth. The date of the first DES-exposed live birth was the study entry date; unexposed women were matched within ±2 years to the DES-exposed women's birth dates, and were assigned the same date of study entry as the exposed woman to whom they were matched. Active follow-up of the WHS cohort was implemented in the early 1980s and continued intermittently through 1989.

The second cohort consists of women who participated in the Dieckmann Study, a placebo-controlled clinical trial of the effects of DES on pregnancy losses. The trial was conducted in the early 1950s at the University of Chicago, and enrolled women who were 6–20 weeks pregnant. The cumulative dose of DES tested was 11–12 g. For these women, the date of pregnancy outcome was the study entry date. Participants were re-contacted for follow-up in 1976. In both the WHS and the Dieckmann cohorts, DES exposure (or lack thereof) was documented in the medical record, the occurrence of cancer was confirmed by medical record, and cause of death was verified by death certificate.

Follow-up of the combined cohort was implemented by the National Cancer Institute (NCI) in 1992, at which time intense tracing efforts were used to locate the women who previously participated in the WHS and Dieckmann studies. In conjunction with the 1994 data collection phase, we obtained either a questionnaire (including proxy questionnaires) or a death certificate for 6495 (84%) of the 7758 women initially ascertained for the WHS and Dieckmann studies. Deaths occurring through the 1994 data collection phase were ascertained by death certificate; subsequently, mortality and cause of death were ascertained through the National Death Index. For a subset of the WHS women (those initially ascertained through the Mayo Clinic), the underlying cause of death was determined through the Social Security Death Index. Follow-up through both sources continued through 1 January 2000.

For the present analysis, the outcomes were all-cause mortality, cause-specific mortality, including overall cancer death, and cause-specific cancer death. Diseases were classified by trained nosologists according to the International Classification of Diseases (ICD), using both ICD-9 (Hart, 2000) and ICD-10 (WHO, 2003). Outcomes represented by fewer than 10 events were classified as ‘other’, except when the outcome was potentially relevant to DES exposure (i.e., vulvar/vaginal and other gynaecologic cancers).

The analyses are based on 7675 of the 7758 women from the original cohorts (83 women were omitted owing to lack of information on study entry date). Covariate information was available for 7062 (92%) women, based predominantly on the 1994 questionnaire responses, or on earlier questionnaire responses when necessary. Cox proportional hazards models (Cox, 1972), with time since study entry as the timescale, were used to estimate mortality rate ratios associated with DES exposure. The women contributed person-time from the date of study entry until the date of death, or 1 January 2000, whichever occurred first. For the outcomes of ovarian, uterine, and cervical cancer, separate analyses censored follow-up at the date of hysterectomy. In general, the analyses were conducted in the combined cohorts; in the presence of interaction between DES and cohort, the outcomes are reported separately for the WHS and Dieckmann cohorts. To assess departure from the proportional hazards assumption, we assessed log–log survival plots and formally tested interaction terms between DES and time (since study entry) in relation to the study outcomes.

Year of birth and study entry year were covariates in all models. Additional covariates, including education (in years; 0–8, 9–12, 13–16, ⩾17), smoking (ever, never), and body mass index (BMI) (kg m^−2^) (⩽21, 21–23, 24–27, ⩾28) were considered when available and appropriate for specific mortality outcomes. Breast cancer models contained terms for family history of breast cancer, BMI, age at first pregnancy (<20, 20–24, 25–29, 30–39, ⩾40), and parity (1–2, 3–4, ⩾5); ovarian cancer analyses were adjusted for parity. We found no evidence of confounding by these variables or by cohort (RR estimates changed less than 10%); consequently, the RR shown in the tables are adjusted for birth year and study entry year. Missing covariate data were included in models by use of indicator variables.

## RESULTS

Table 1 shows the current status of the 7675 women on whom our analyses are based. Of these, 2237 (29.1%) (1156 exposed, 1081 unexposed) were known to have died and cause of death was determined for 2193 (98.0%) (1128 exposed, 1065 unexposed).

Exposed and unexposed women in the combined cohort were of comparable age at study entry. Education, BMI, parity, and smoking history, when available, were similar for the exposed and unexposed (Table 2).

In the combined cohort, the RR for DES in relation to all-cause mortality was 1.06 (95% CI=0.98–1.16) (Table 3). When breast cancer deaths were not counted as outcomes, the RR was 1.05 (95% CI=0.96–1.14) in the combined cohort, 1.03 (95% CI=0.94–1.13) in WHS, and 1.12 (95% CI=0.91–1.39) in the Dieckmann cohort. After additional adjustment for BMI and smoking, and with breast cancer omitted, the RR was 1.11 (95% CI=1.02–1.21) in the combined cohort; the findings were identical for the individual cohorts.

The combined grouping of cerebrocardiovascular disease accounted for about one-third (33.8%) of all deaths, but there was little evidence that DES increased overall cerebrocardiovascular mortality or mortality from cardiovascular, cerebrovascular, or other vascular conditions.

The slightly elevated RR for DES in relation to mortality owing to infectious disease, neuropsychiatric conditions, Alzheimer's disease, digestive diseases, and diabetes were compatible with chance. A moderately strong and statistically significant association was observed between DES exposure and deaths owing to kidney/urinary tract disease (RR=2.39; 95% CI=1.14–4.99), and the RR was 2.57 (95% CI=1.23–5.39) after further adjustment for smoking. Mortality from violence/accident was similar for the exposed and unexposed women. There was little evidence of an association between DES and noncancer mortality classified as ‘other’; RR=0.90 (95% CI=0.62–1.29). Inverse associations were observed for pulmonary and liver disease mortality in relation to DES exposure, but were consistent with chance, and the results were similar after adjustment for smoking (data not shown).

Overall, 37.7% of deaths were owing to cancer (Table 3). In the combined cohort, the RR for overall cancer mortality was 1.07 (95% CI=0.94–1.23). In analyses that omitted the breast cancer outcomes, the RR was 1.02 (95% CI=0.88–1.19) in the combined cohort, 0.96 (95% CI=0.81–1.14) in the WHS, and 1.37 (95% CI=0.94–2.00) in the Dieckmann cohort. The interaction between DES exposure and cohort in relation to overall cancer mortality was not statistically significant (*P*=0.11). After further adjustment for BMI and smoking, and with breast cancer deaths omitted, the RR for overall cancer mortality was 1.09 (95% CI=0.94–1.28) in the combined cohort, 1.06 (95% CI=0.90–1.26) in WHS, and 1.35 (95% CI=0.92–1.97) in the Dieckmann cohort.

In the combined cohort, the RR was 1.27 (95% CI=0.96–1.69) for the association between DES and breast cancer death; after further adjustment for family history of breast cancer, BMI, age at first pregnancy, and parity, the RR was 1.38 (95% CI=1.03–1.85). The RR was 1.12 (95% CI=0.80–1.55) in the WHS cohort and 1.82 (95%=1.04–3.18) in the Dieckmann cohort, but the interaction between DES and cohort in relation to breast cancer mortality was not statistically significant (*P*=0.15). The association with breast cancer mortality did not change over time; the RR was 1.31 and 1.25, respectively, for less than 30 years, and 30 or more years since exposure.

Diethylstilbestrol was not associated with an increased risk of death due to ovarian cancer (RR=0.87; 95% CI=0.51–1.46); the RR was essentially the same after adjustment for parity, and when follow-up was censored at hysterectomy. The RR were 1.48 (95% CI=0.53–4.17) and 0.25 (95% CI=0.05–1.18), respectively, for the associations with uterine and cervical cancer mortality, and both RR were materially unchanged after adjustment for smoking and when follow-up was censored at hysterectomy. There was no association with vulvar/vaginal cancer (RR=1.00; 95% CI=0.20–4.93).

The RR suggested elevated risk of death from lung, urinary, and head and neck cancer, as well as non-Hodgkin's lymphoma, melanoma, and leukaemia, but confidence intervals were wide and included the null value. Further adjustment for smoking did not materially change the RR of the cancers known to be associated with that exposure. The inverse associations suggested for brain cancer, cancer of the upper GI tract, and ‘other’ cancer were also imprecise. The inverse association with death from multiple myeloma was statistically significant, RR=0.18 (95% CI=0.04–0.82), and was unchanged after adjustment for smoking. The data provided no evidence that DES was related to death from colorectal or other GI cancers.

## DISCUSSION

Our results are based on the largest mortality study to date of women with documented exposure to DES during pregnancy, with over 2000 deaths, of which 800 were from cancer and nearly 200 from breast cancer.

Our data suggest that DES is associated with a small elevation of all-cause mortality. The finding may reflect residual confounding by a lifestyle correlate, although the RR was somewhat greater in the Dieckmann cohort, which consists of women who were enrolled in a clinical trial of DES and who received high doses of DES. There was no increase of cerebrocardiovascular deaths, and the suggested increased risk of vascular deaths was consistent with chance. Clinical trial results from the Women's Health Initiative (WHI) suggest increased risks of cerebrocardiovascular events, including stroke and thromboembolic events, in postmenopausal women using equine oestrogens, but the effects may be limited to current or recent use (Women's Health Initiative, 2004). Also, WHI participants were 50–70 years of age at enrolment, whereas women in our study were in their childbearing years at the time of DES use and at lower risk of cerebrocardiovascular outcomes. Current use of menopausal oestrogen may also increase risk of cognitive impairment (Shumaker *et al*, 2004), but our data showed no association with mortality from psychiatric disease or Alzheimer's disease. Although the data indicated a moderately elevated mortality for kidney/urinary disease and an inverse association with ‘other’ noncancer mortality, both of which were statistically significant, we assessed a large number of outcomes, and these findings may represent false positives. We are unaware of human evidence linking DES to kidney/urinary disease, although DES-induced structural and functional changes in the renal tissue of rats have been reported (Onarglioglu *et al*, 1998).

The increased risk of breast cancer death observed in the combined cohort is consistent with the large follow-up study of DES in relation breast cancer mortality (Calle *et al*, 1996) and with our previous findings (Titus-Ernstoff *et al*, 2001). An early analysis of the Dieckmann data suggested a moderately strong association with breast cancer mortality (RR=2.89; 95% CI=0.99–8.47) (Clark and Portier, 1979), and our finding in the combined cohort was largely owing to the elevated mortality in the Dieckmann group. The association with breast cancer mortality in the Dieckmann cohort is noteworthy because, unlike the WHS, DES exposure was in a clinical trial, and was not owing to a history of pregnancy complications, indicating an aberrant hormonal milieu or specialised obstetrics care, which might be correlated with lifestyle characteristics.

Women participating in the Dieckmann trial received especially high doses of DES resulting in a cumulative dose of 11–12 g over the course of the pregnancy. Based on studies involving women treated during obstetrics care (Hadjimichael *et al*, 1984), doses given to pregnant women in the WHS were probably much lower (about 1.1 g cumulative). Possibly, the stronger association between DES and breast cancer mortality observed in the Dieckmann study reflects the higher doses of DES administered to women in that cohort and/or the clinical trial design, which should eliminate confounding. However, a study examining dose in relation to breast cancer risk found no evidence of dose response (Hadjimichael *et al*, 1984), and our previous study of breast cancer incidence in the combined cohort showed similar risks for WHS and Dieckmann women (Titus-Ernstoff *et al*, 2001). Some studies of breast cancer incidence, including two previous studies based on the WHS and Dieckmann cohorts, suggested possible latency effects (Bibbo *et al*, 1978; Colton *et al*, 1993), but others did not (Greenberg *et al*, 1984; Hadjimichael *et al*, 1984; Titus-Ernstoff *et al*, 2001), including a large study conducted in Connecticut (Hadjimichael *et al*, 1984). Consistent with previous reports (Hadjimichael *et al*, 1984; Calle *et al*, 1996), we found no evidence that breast cancer mortality differed according to time since exposure.

Although findings in the Dieckmann cohort suggested a small increased risk of overall cancer mortality, even after removing breast cancer deaths, the results were consistent with chance, as were the findings for the combined cohort. We also found little evidence that DES was associated with specific cancers other than breast cancer. Previous studies of endometrial cancer and DES during pregnancy or as hormone therapy have produced conflicting results; one study suggested a decreased risk (Hadjimichael *et al*, 1984), whereas others showed an increased risk (Hoover *et al*, 1976; Autunes *et al*, 1979). In our data, the modestly elevated mortality rate ratio for uterine cancer was compatible with chance. We also found no evidence that DES exposure was associated with ovarian or cervical cancer. Others have suggested increased risk of ovarian (Hoover *et al*, 1977; Hadjimichael *et al*, 1984) and possibly cervical cancer (Hadjimichael *et al*, 1984), but case numbers were small. We also found no indication that DES use during pregnancy was associated with vaginal/vulvar cancers, tumours that occur in women exposed to DES *in utero* (Herbst *et al*, 1971). We have no explanation for the strong inverse association with multiple myeloma, but we assessed numerous outcomes, and the finding may be a false positive.

The size of the combined cohort, the large number of outcomes, and the documented use of DES are strengths of our study. Diethylstilbestrol doses are known for the Dieckmann women, most of whom received a high cumulative dose, but are unknown for the WHS participants, precluding assessment of risk according to dose. Although we had limited information on potential confounders, some data were available for major health indicators such as BMI and smoking, and breast cancer risk factors were available for most women. The possibility of incomplete mortality ascertainment is a limitation of all studies relying on NDI searches. Similar proportions of the exposed or unexposed women in the initial cohort were lost to follow-up as of the 1994 data collection phase (6.8% exposed, 9.3% unexposed), and cause of death was ascertained for 97.6% of the exposed and 98.5% of unexposed decedents. Although incomplete ascertainment may have attenuated some associations, it seems unlikely that ascertainment would be associated with DES exposure; so our results should not be biased away from the null.

In summary, our findings indicate that women given DES during pregnancy experienced a slight but statistically significant elevation in all-cause mortality after adjusting for covariates and omitting breast cancer deaths. Our data did not show an elevated risk of mortality from cancer overall or from gynaecological cancers. However, an increased risk of breast cancer mortality was evident, particularly in the Dieckmann women, who received high doses of DES during pregnancy.

## Figures and Tables

**Table 1:** Status of the original cohorts

	**Women's Health Study**		**Dieckmann Study**		
	**Exposed**	**Unexposed**	**Exposed**	**Unexposed**	**Total**
*Status of cohorts*					
Initial members^a^	3031	3014	826	804	7675
Ever followed	2885	2816	693	668	7062
Deceased	931	900	225	181	2237
Cause of death known	909	887	219	178	2193
1994 Quest returned	2079	2011	425	430	4945
Lost to follow-up^b^	112	180	150	181	623

a Of 7758 women in the initial cohort, 83 WHS women were removed owing to an unknown study entry date.

b At the time of the 1994 follow-up.

**Table 2:** Characteristics of women in the combined cohort, by DES exposure status

	**Exposed**,	**Unexposed**,
	***N*=3857**,	***N*=3818**,
**Factor**	**number (%)**	**number (%)**
*Age at study entry*		
<25	1101 (28.6)	1087 (28.5)
25–29	1362 (35.3)	1344 (35.2)
30–34	832 (21.6)	867 (22.7)
⩾35	562 (14.6)	520 (13.6)
		
Mean age at study entry	28.1	28.1
		
*Years of education* a		
0–8	164 (6.7)	188 (8.0)
9–12	1205 (49.0)	1139 (48.5)
13–16	899 (36.5)	838 (35.7)
⩾17	193 (7.8)	182 (7.8)
Missing	1396	1471
		
Mean years of education	13.0	12.9
		
*Body mass index* b		
<21	365 (13.1)	357 (13.1)
21–23	1040 (37.3)	935 (34.4)
24–27	892 (32.0)	957 (35.2)
⩾28	493 (17.7)	469 (17.3)
Missing	1067	1100
		
Mean body mass index	24.4	24.5
		
*Ever smoked*±±		
No	1700 (49.8)	1665 (51.0)
Yes	1712 (50.2)	1600 (49.0)
Missing	445	553

BMI=body mass index; WHS=Women's Health Study.

a Education missing for 100% of Dieckmann women and 20% of WHS women.

b BMI missing for 40% of Dieckmann women and 25% of WHS women. The BMI was based on weight at age 50 for WHS women and on current weight at most recent questionnaire response for the Dieckmann cohort.

±±Smoking missing for <1% of Dieckmann women and 16% of WHS.

**Table 3:** Mortality rate ratios for the combined cohort, by DES exposure status

	**Number of deaths**		
**Cause of death**	**Exposed**	**Unexposed**	**RR (95% CI)^a^**
All mortality	1156	1081	1.06 (0.98–1.16)
			
*Cerebrocardiovascular*	383	373	1.02 (0.88–1.18)
Cardiovascular	273	276	0.98 (0.83–1.16)
Cerebrovascular	80	74	1.08 (0.78–1.47)
Other vascular	30	23	1.29 (0.75–2.23)
Infectious	60	43	1.39 (0.94–2.06)
*Neuropsychiatric*	54	42	1.29 (0.86–1.92)
Alzheimer's	8	7	1.17 (0.42–3.22)
Pulmonary	37	46	0.80 (0.52–1.24)
Violence/accident	39	38	1.02 (0.65–1.60)
Digestive	27	20	1.34 (0.75–2.40)
Diabetes	26	20	1.30 (0.72–2.32)
			
Liver	13	22	0.59 (0.30–1.16)
Kidney/urinary	24	10	2.39 (1.14–4.99)
Other (noncancer)	55	61	0.90 (0.62–1.29)
			
*Cancer mortality*	438	406	1.07 (0.94–1.23)
Breast	110	86	1.27 (0.96–1.69)
Lung	96	81	1.19 (0.88–1.60)
Colorectal	42	44	0.95 (0.62–1.45)
Upper GI	9	13	0.69 (0.30–1.61)
Other GI	34	34	1.00 (0.62–1.60)
Ovary	26	30	0.87 (0.51–1.46)
Uterine	9	6	1.48 (0.53–4.17)
Cervix	2	8	0.25 (0.05–1.18)
Vulvar/vaginal	3	3	1.00 (0.20–4.93)
Urinary	15	11	1.36 (0.62–2.96)
Brain	10	12	0.83 (0.36–1.93)
Non-Hodgkin's lymphoma	25	16	1.56 (0.83–2.92)
Leukaemia	15	9	1.66 (0.73–3.80)
Head and neck	9	5	1.80 (0.60–5.36)
Melanoma	7	5	1.39 (0.44–4.37)
Multiple myeloma	2	11	0.18 (0.04–0.82)
Other cancer	24	32	0.75 (0.44–1.27)

GI=gastrointestinal.

a Adjusted for birth year and year of study entry.

## References

[bib1] Autunes CMF, Stolley PD, Rosenshein NB, Davies JL, Tonascia JA, Brown C, Burnett L, Rutledge A, Pokempner M, Garcia R (1979) Endometrial cancer and estrogen use. Report of a large case-control study. N Engl J Med 300: 9–13 213722 10.1056/NEJM197901043000103

[bib2] Beral V, Colwell L (1980) Randomised trial of high doses of stilboestrol and ethisterone in pregnancy: long term follow-up of mothers. BMJ 281: 1098–1101 7000292 10.1136/bmj.281.6248.1098 PMC1714571

[bib3] Bibbo M, Haenszel WM, Wied GL, Hubby M, Herbst AL (1978) A twenty-five-year follow-up study of women exposed to diethylstilbestrol during pregnancy. N Engl J Med 298: 763–767 628409 10.1056/NEJM197804062981403

[bib4] Calle EE, Mervis CA, Thun MJ, Rodriguez C, Wingo PA, Heath Jr CW (1996) Diethylstilbestrol and risk of fatal breast cancer in a prospective cohort of US women. Am J Epidemiol 144: 645–652 8823060 10.1093/oxfordjournals.aje.a008976

[bib5] Clark LG, Portier KM (1979) Diethylstilbestrol and the risk of cancer. N Engl J Med (ltr) 300: 263–264 10.1056/NEJM197902013000519 759877

[bib6] Colton T, Greenberg ER, Noller K, Resseguie L, Van Bennekom C, Heeren T, Zhang Y (1993) Breast cancer in mothers prescribed diethylstilbestrol in pregnancy. J Am Med Assoc 269: 2096–2100 8468763

[bib7] Cox DR (1972) Regression models and life tables. J R Stat Soc B 34: 187–220

[bib8] Dieckmann WJ, Davis ME, Rynkiewicz LM, Pottinger RE (1953) Does the administration of diethylstilbestrol during pregnancy have therapeutic value? Am J Obstet Gynecol 66: 1062–1081 13104505 10.1016/s0002-9378(16)38617-3

[bib9] Greenberg ER, Barnes AB, Resseguie L, Barrett JA, Burnside S, Lanza LL, Neff RK, Stevens M, Young RH, Colton T (1984) Breast cancer in mothers given diethylstilbestrol in pregnancy. N Engl J Med 311: 1393–1398 6493300 10.1056/NEJM198411293112201

[bib10] Hadjimichael OC, Meigs JW, Falcier FW, Thompson WD, Flannery JT (1984) Cancer risk among women exposed to exogenous estrogens during pregnancy. J Natl Cancer Inst 73: 831–834 6592380

[bib11] Hart AC (ed) (2000) ICD-9 Code Book. Reston, VA: St Anthony Publishing

[bib12] Herbst AL, Ulfelder H, Poskanzer DC (1971) Adenocarcinoma of the vagina. Association of maternal stilbestrol therapy with tumor appearance in young women. N Engl J Med 284: 878–881 5549830 10.1056/NEJM197104222841604

[bib13] Hoover R, Fraumeni Jr JF, Everson R, Myers MH (1976) Cancer of the uterine corpus after hormonal treatment for breast cancer. Lancet 1: 885–887 58149 10.1016/s0140-6736(76)92099-7

[bib14] Hoover R, Gray LA, Fraumeni Jr JF (1977) Stilboestrol (Diethylstilbestrol) and the risk of ovarian cancer. Lancet 2: 533–534 95735 10.1016/s0140-6736(77)90667-5

[bib15] Newbold RR (1993) Gender-related behavior in women exposed prenatally to diethylstilbestrol. Environ Health Perspect 101: 208–213 8404755 10.1289/ehp.93101208 PMC1519765

[bib16] Noller KL, Fish CR (1974) Diethylstylbestrol usage: its interesting past, important present, and questionable future. Med Clin N Am 58: 793–810 4276416 10.1016/s0025-7125(16)32122-8

[bib17] Onarglioglu B, Gunay Y, Goze I, Koptagel E (1998) Renal ultrastructural alterations following administration of diethylstilbestrol. Tr J Med Sci 28: 369–374

[bib18] Shumaker SA, Legault C, Kuller L, Rapp SR, Tal L, Lane DS, Fillit H, Stefanick ML, Hendrix SL, Lewis CE, Masaki K, Coker LH (2004) Conjugated equine estrogens and incidence of probably dementia and mild cognitive impairment in postmenopausal women. J Am Med Assoc 291: 2947–2958 10.1001/jama.291.24.2947 15213206

[bib19] Swyer GIM, Law RG (1954) An evaluation of the prophylactic ante-natal use of stilboestrol. Preliminary report. J Endocrinol 10: vi

[bib20] Titus-Ernstoff L, Hatch EE, Hoover RN, Palmer J, Greenberg ER, Ricker W, Kaufman R, Noller K, Herbst AL, Colton T, Hartge P (2001) Long-term cancer risk in women given diethylstilbestrol (DES) during pregnancy. Br J Cancer 84: 126–133 11139327 10.1054/bjoc.2000.1521 PMC2363605

[bib21] Vessey MP, Fairweather DV, Norman-Smith B, Buckley J (1983) A randomized double-blind controlled trial of the value of stilboestrol therapy in pregnancy: long-term follow-up of mothers and their offspring. Br J Obstet Gynecol 90: 1007–1017 10.1111/j.1471-0528.1983.tb06438.x 6357269

[bib22] Women's Health Initiative Steering Committee (2004) Effects of conjugated equine estrogen in postmenopausal women with hysterectomy. J Am Med Assoc 291: 1701–1712 10.1001/jama.291.14.1701 15082697

[bib23] World Health Organization (2003) ICD-10 Code Book. International Classification of Diseases. US Natl Center Health Stat

